# Investigating the in vitro photothermal effect of green synthesized apigenin‐coated gold nanoparticle on colorectal carcinoma

**DOI:** 10.1049/nbt2.12016

**Published:** 2021-02-22

**Authors:** Seyed Mohammad Amini, Elham Mohammadi, Shaghayegh Askarian‐Amiri, Yaser Azizi, Ali Shakeri‐Zadeh, Ali Neshastehriz

**Affiliations:** ^1^ Radiation Biology Research Center Iran University of Medical Sciences (IUMS) Tehran Iran; ^2^ Physiology Research Center Iran University of Medical Sciences (IUMS) Tehran Iran; ^3^ Department of Physiology School of Medicine Iran University of Medical Sciences Tehran Iran; ^4^ Medical Physics Department School of Medicine Iran University of Medical Sciences (IUMS) Tehran Iran

## Abstract

Applying toxic chemical to the synthesis of stable gold nanoparticles is one of the limitations of gold nanoparticles for therapeutic applications such as photothermal therapy. Plant compounds such as apigenin (API) with therapeutic potential can be applied in the synthesis of gold nanoparticles. API‐coated gold nanoparticles (Api@AuNPs) with an average size of 19.1 nm and a surface charge of −4.3 mV have been synthesized by a simple and efficient technique. The stability of Api@AuNPs in the biological environment was verified through UV‐Vis spectroscopy. Based on Raman and FTIR spectroscopy analysis, chemical binding of API on the surface of Api@AuNPs through hydroxyl and carbonyl functional groups was found to be the main reason for the stability of the Api@AuNPs in comparison with citrate‐coated gold nanoparticles (Cit@AuNPs). The synthesized Api@AuNPs do not cause major toxic effects up to 128 ppm. Api@AuNP‐mediated photothermal therapy leads to the indiscriminate eradication of almost half of both mouse fibroblastic (L929) and colorectal cancer (CT26) cells. Flow‐cytometry analysis revealed that the cell death mechanism is mainly apoptosis. In the apoptosis triggered cell death in photothermal treatment, Api@AuNPs are preferred over commonly used gold nanoparticles in photothermal treatments which mostly trigger the necrosis cell death pathway.

## 1 INTRODUCTION

Developing new nanomaterials for cancer treatment is attracting attention worldwide. Gold nanomaterials with unique properties such as high proton number (Z), proper biocompatibility, and characteristic surface plasmon resonance (SPR) peak were applied for the improvement of different types of cancer treatment such as chemotherapy, radiation therapy, hyperthermia, and photodynamic therapy [1]. Photothermal therapy with gold nanoparticles is one of the newest methods for cancer treatment. This technique has attracted many researchers in the field of cancer treatment. Previous studies demonstrate that heat generated by nanoparticles under laser irradiation leads to necrosis of the cells that may lead to undesirable inflammatory responses [2].

For the preparation of monodispersed and stable gold nanoparticles in different sizes, the wet chemical method is the main procedure for nanoparticle preparation that needs some organic or inorganic chemicals as a reducing agent and stabilizer. Some of these chemicals such as borohydride derivatives are dangerous toxic materials [3]. The toxicity of some other chemicals such as sodium citrate or mercapto compounds are under scrutiny [4]. Plant extracts contain various phytochemicals such as aldehydes, flavonoids, terpenoids, acids, ketones that could be applied for the synthesis of metal and metal oxide nanoparticles [5]. However, most of the metal nanoparticles that have been synthesized through plant extracts are not only heterogeneous in size and shape but also contain various chemicals with possible unwanted side effects. Applying specific plant's secondary metabolites with metal ion reducing capabilities such as flavonoids or phenolic acids were considered for gold nanoparticle preparation based on wet chemical methods instead of dangerous chemicals [6, 7].

**FIGURE 1:**
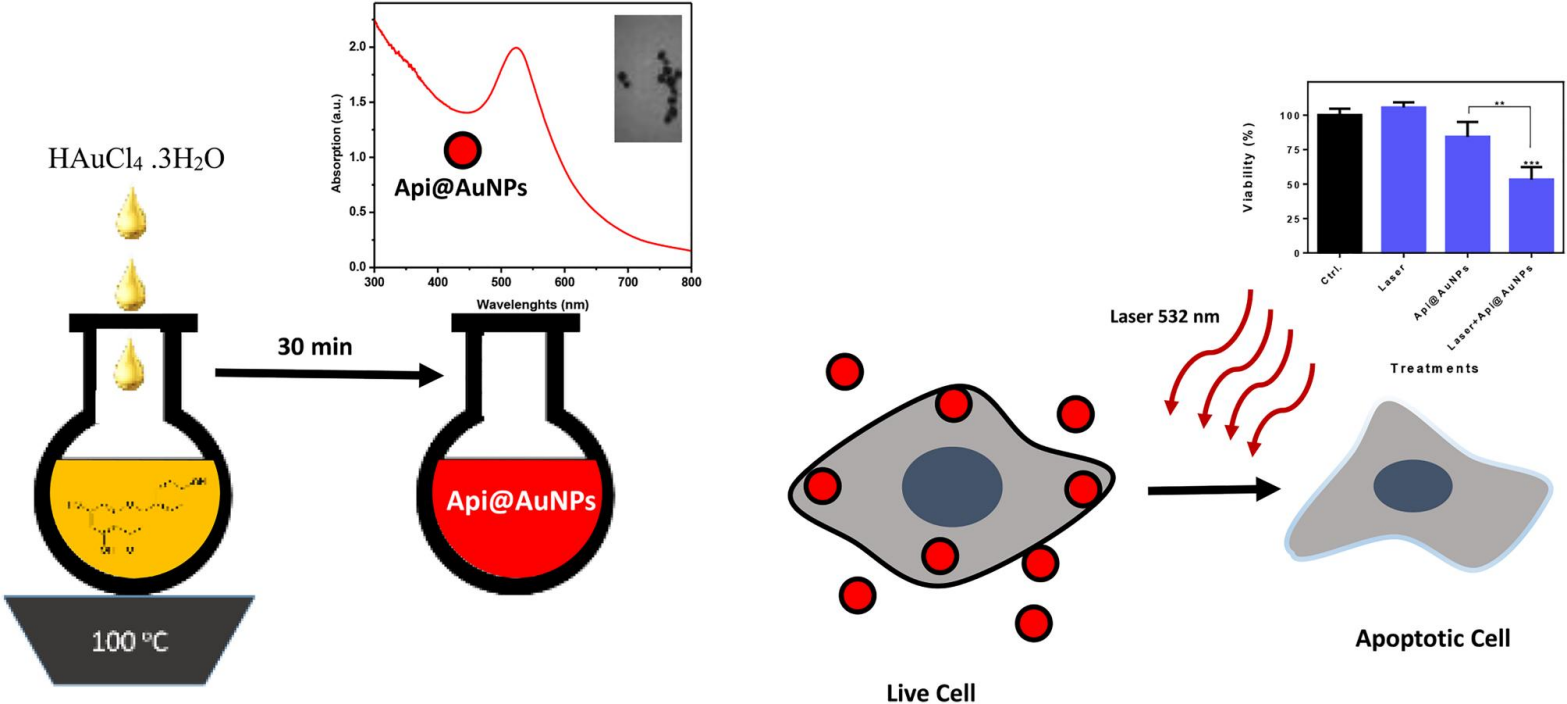
A schematic view of the synthesis and application of Api@AuNPs in the in vitro photothermal therapy

Apigenin (API) is a flavonoid that representing anti‐inflammatory and anticancer effects. Through the autophagy and apoptosis process, it can suppress cancer cells [8]. Rajendran et al. claimed that API‐coated gold nanoparticles (Api@AuNPs) didn't show any effect on healthy cells and only cause apoptosis in cancerous cells [9]. Besides the therapeutic function of API, Api@AuNPs can increase the efficiency of radiation therapy because of the high Z number of gold [10]. In this study, the parameters for synthesis and characterization of Api@AuNPs are improved and subsequently in vitro photothermal effect of these structures was investigated for the first time. A schematic view of the manuscript is provided in Figure 1. The synthesized nanoparticles are spherical and well covered by the phenolic compound and represent high biocompatibility for both cells. In vitro photothermal therapy treatment leads to the eradication of almost half of the cells through apoptosis‐mediated cell death.

## 2 MATERIAL AND METHODS

### 2.1 Chemical

Trachloroauric acid trihydrate 99.5% (HAuCl_4_ .3H_2_O), Potassium carbonate (K_2_CO_3_), dimethyl sulfoxide (DMSO), hydrochloric acid (ACS reagent, 37%), and nitric acid (ACS reagent, 65%) were purchased from Merck chemicals company (Darmstadt, Germany). API was purchased from Shaanxi Huike Botanical Development Co. Ltd. RPMI‐1640 culture medium (Biosera, UK), which supplemented by 1% penicillin/streptomycin (Hyclone, Utah, USA) and 10% bovine serum albumin (Hyclone, Utah, USA), was used for in vitro studies. Thiazolyl blue tetrazolium bromide (MTT) was purchased from Sigma‐Aldrich (St.Luis.MO). Deionized water that obtained from Milli‐Q Plus 185 water purification system (Millipore, Bedford, MA) (resistivity of 18.3 MΩcm) was used for all aqueous solution. All aqueous solutions were provided with DI water. Glassware were soaked with Aqua Regia and rinsed thoroughly with DI water.

### 2.2 Synthesis and characterization

Citrate‐coated gold nanoparticles (Cit@AuNPs) were synthesized based on our previous studies [11, 12]. Api@AuNPs were synthesized based on Rajendran et al. report [9] with few important modifications. 150 µl of 20 mM solution of API in DMSO is added to 10 ml of hot boiled DI water. The pH of the water was set in the range of 10 by the addition of K_2_CO_3_ (300 mM) before adding API. 5 ml of HAuCl_4_ .3H_2_O (2.5 mM) was added subsequently and the solution was left for 30 min. The appearance of a shiny red colour is an indication of gold nanoparticle formation. Unreacted gold ion and API molecules were removed through a series of centrifugation and decantation at 20,000 rpm. In the final round, the Api@AuNPs were dispersed with a small volume of deionized water to obtain higher concentrations of the Api@AuNPs. The final concentration of gold nanoparticles was assessed using ICP‐AES analysis. Transmission electron microscope (Zeiss EM 900, Germany) was applied for the size and morphology investigation of synthesized nanoparticles. Double beam UV‐visible absorption spectrophotometer (SPEKOL 2000, Analytik Jena, UK) with 1 cm of optical path length quartz cuvette was used for acquiring spectrum for different analysis. Nano‐flex device was applied for Zeta potential (Zeta‐check, Microtrac, Germany) and hydrodynamic diameter (DLS, NANO‐flex Particle Sizer Germany) analysis. Nicolet Avatar 360 FTIR (Thermo Scientific, Courtaboeuf, France) was used for Fourier transform infrared spectroscopy (FTIR) studies and for further investigation of Raman scattering spectra of Api@AuNPs and API, specimens were recorded by AvaSpec‐ULS2048x64TEC, Avantes Spectroscope (Apeldoorn, Netherlands). For laser treatments of cells, a continuous‐wave laser system at a wavelength of 532 nm, was obtained from Nanobon Company (Tehran, Iran).

### 2.3 Cell analysis

Our investigations were performed at the mouse fibroblastic (L929) and colorectal cancer (CT26) cell lines. During the experiments, cells were incubated in a humidified atmosphere at 37°C and 5% CO_2_ with RPMI 1640 growth medium supplemented with 10% fetal bovine serum and 1% penicillin/streptomycin. To examine the cytotoxicity of Api@AuNPs, 10,000 cells were seeded into each well of 96‐well plate (well bottom area = 0.32 cm^2^) and after 24 h the cell culture was succeeded with fresh cell culture contained different concentrations of nanoparticles. After another 24 h incubation, cells were washed with PBS before MTT assay. For photothermal studies, after treatment of the cells with 128 μg/ml Api@AuNPs wells have been washed with PBS and incubated for another 24 h. The selected wells were irradiated with continuous‐wave laser (Power density: 0.5 W/cm^2^, 5 or 3 min), and after that cell, the medium was substituted and the cells were incubated for another 24 h. Before the MTT assay, cells were washed with PBS.

### 2.4 MTT assay

After different treatments, cells have been washed with PBS carefully for removing free Api@AuNPs. Then cells were incubated with 100 ml, 0.5 mg/ml solution of MTT in PBS for 2–4 h that was then replaced with 100 ml DMSO. The absorbance value of each well was read at a wavelength of 570 nm.

### 2.5 Flow cytometry studies

The flow cytometry was performed to compare the apoptotic effects of different treatments with applying Annexin‐V‐fluorescein isothiocyanate (FITC)‐propidium iodide (PI) (eBioscience, San Diego, CA, USA) kite. Similar to PTT experiments in MTT assay, cells have been seeded, treated by Api@AuNPs, and irradiated by laser on consecutive days. Then cells were trypsinized, collected, and suspended 1× buffer at a concentration of 1 × 10^6^ cells/ml. In the next step, 5 ml of PI and 5 ml of Annexin V–FITC were poured into the above 1× binding buffer, and the cells were incubated in dark at room temperature for another 20 min. Subsequently, the quantitative interpretation of cell apoptosis and necrosis were conducted directly by flow cytometry.

### 2.6 Statistical analysis

Data were represented as mean ± SD. The one‐way analysis of variance (ANOVA) was applied to compare the significance of the experimental data. Statistical significance, α, was set to *p* < 0.05. Data were analysed and plotted with Origin 2015 (OriginLab Co., USA), and GraphPad Prism 6 (GraphPad Software, San Diego, CA).

## 3 RESULTS AND DISCUSSION

### 3.1 Synthesis and characterization

**FIGURE 2:**
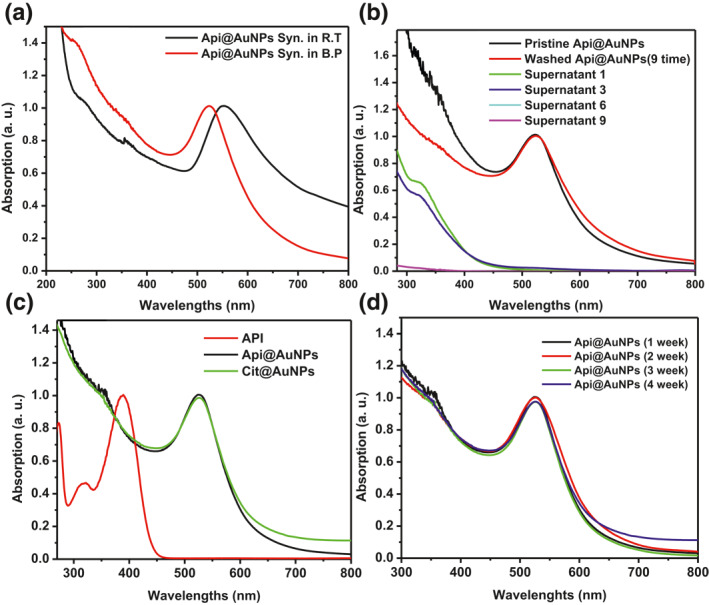
UV‐Vis spectroscopic analysis of Api@AuNPs. UV‐Vis spectrums of synthetized Api@AuNPs in room and boiling temperature (a). The spectrums of pristine and washed Api@AuNPs and supernatant of each washing step (b). Api@AuNPs, API (0.3 mM) and Cit@AuNPs spectra (c). The stability of washed Api@AuNPs in a period of 4 weeks (d)

Synthesis and stability of Api@AuNPs were investigated by UV‐Vis Spectrum. For the synthesis procedure, the boiling temperature is a critical factor which not noted by Rajendran et al. [9]. The SPR peaks of Api@AuNPs that synthesized in room temperature is 540 nm which could indicate an average size of 80 nm. However, for synthesized Api@AuNPs in the boiling point, the SPR peak appears at 523 nm (Figure 2a) which correlates to smaller sizes based on data that have been provided by Haiss et al. [13]. After synthesis of Api@AuNPs, removing free API and Au ions is essential for the purification of the final product. After nine rounds of centrifugation and decantation, no trace of API or gold absorbance was observed in the supernatant UV‐Vis spectrum. In previous supernatants, the optical absorption of these substances was observed in the corresponded spectra (Figure 2b).

The characteristics values of Api@AuNPs and free API were compared with well‐known Cit@AuNPs which has been widely studied by many researchers. Synthesis of spherical Api@AuNPs and Cit@AuNPs was verified by the presence of characteristic surface plasmon resonance in the UV‐Vis absorption spectra. λ max of API was observed in 390 and 273 nm (Figure 2c); these bands are the representation of A and B ring of the API [14].

In this experiment, the stability of the aqueous solution of Api@AuNPs was studied through UV‐Vis spectroscopy. UV‐Vis spectra of an aqueous solution of Api@AuNPs were recorded after 4 weeks of the preparation. No shift or broadening of SPR peaks was observed which indicates the stability of synthesized nanoparticles in DI water (Figure 2d).

**FIGURE 3:**
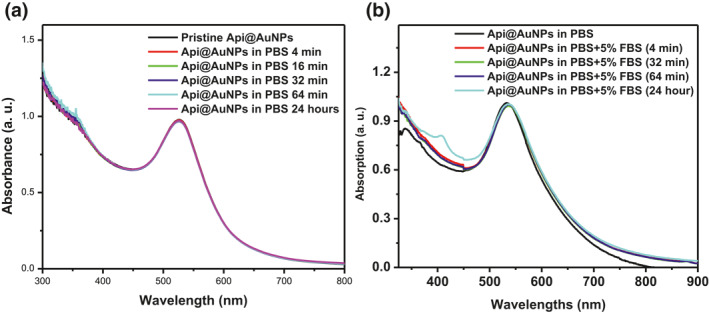
UV‐Vis analysis of Api@AuNPs stability, in the presence of PBS for 24 h (a), the presence of PBS + 5% FBS for 24 h (b)

However, based on our previous study, the presence of different materials such as proteins, salts, and sugars in the cell culture medium can cause the aggregation of Cit@AuNPs nanoparticles [15]. The stability of nanoparticles in a PBS alone and the presence of FBS was studied through UV‐Vis spectroscopy (Figure 3a,b). Api@AuNPs were stable for four weeks in PBS and 24 h in PBS supplemented with 5% FBS for a day. It seems that the stability of the Api@AuNPs is due to the binding of the API to their surface. The surface binding of phytochemicals through the catechol group has also been reported [7, 16].

**FIGURE 4:**
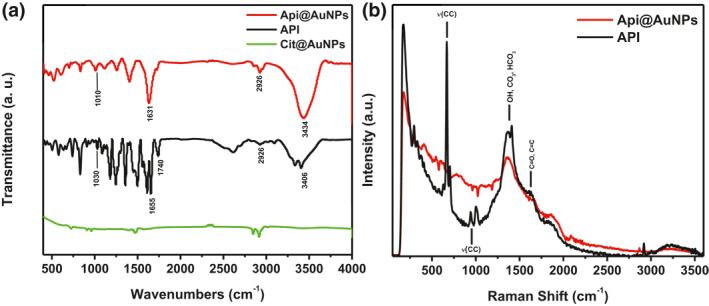
FTIR spectra of Api@AuNPs, API, and Cit@AuNPs (a). Raman spectra of Api@AuNPs and API (b). Many similar peaks were observed for API and Api@AuNPs that have been written on both spectra

We investigate API coating on the surface of nanoparticles through FTIR spectroscopy (Figure 4a). In general, the spectrum of API is similar to Api@AuNPs. However, Cit@AuNPs FTIR spectrum does not represent any significant peaks. Our study shows that several peaks have existed in both spectra that indicate the presence of API on the surface of gold nanoparticles. In brief, 3406 cm^−1^ in the API spectrum and 3434 cm^−1^ in the Api@AuNPs spectrum is assigned for hydrogen bonding of ν O‐H or phenol groups. 2926 cm^−1^ in both samples is assigned for ν C‐H (stretch), 1655 for API and 1631 cm^−1^ for Api@AuNPs representing the C=O stretching vibrations and 1030 cm^−1^ for API and Api@AuNPs is a representation of CCC ring vibrations. Similar FTIR data was provided by Rajendran et al. [9]. C=O stretching bands are observed in the 1740 cm^−1^ for API only. The absence of this band in Api@AuNPs could be an indication of API chemical binding on the surface of gold nanoparticles through the carbonyl functional group. Some IR peaks at 1300–1500 cm^−1^ in the API sample representing the aromatic ring was disappeared in Api@AuNPs sample. However, few aromatic ring peaks appeared at 1150–1400 cm^−1^ which could be considered as a sign for benzoic ring interaction with Au Surface.

Raman Spectra was also applied to investigate the chemical binding of API on the surface of gold nanoparticles (Figure 4b). The broad peak in 1300–1400 cm^−1^ was assigned to bicarbonate and carbonate ion [17]. This peak could represent the C=O that exists in the B ring of API [18]. Also, OH bending (βOH) was attributed to this region in API spectra [19]. The higher intensity of this band for solid API compare to Api@AuNPs could be a proof of interaction of API with gold nanoparticles surface through the hydroxyl or carbonyl group of the molecule. At the right shoulder of this broad peak, another peak appeared around 1600–1620 cm^−1^ that could represent the vibrational mood of C=O or C=C in API the molecule [14, 19]. A distinguished double band at 945 and 1005 cm^−1^ and very strong peaks at 600–700 cm^−1^ was attributed to phenyl ring chain and carbonyl vibrations which were appeared only for solid API samples. The first two bands were also assigned to the ring B trigonal stretch [20]. The disappearances of these peaks in Api@AuNPs could be an indication of the catechol group of apigenin interaction with the gold nanoparticle surface. It has been shown that the catechol group of phenolic compounds can bind to the surface of metal nanoparticles through different configurations [7].

**FIGURE 5:**
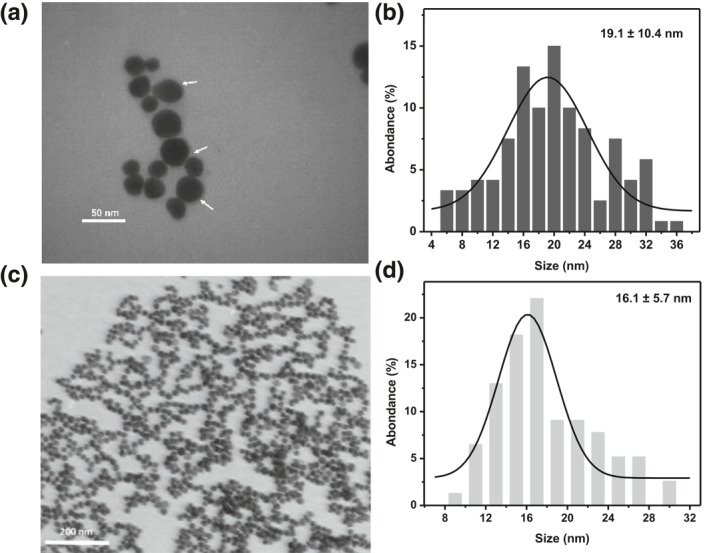
TEM micrographs (a) and the size distribution diagram of Api@AuNPs (b). TEM micrographs (c) and the size distribution diagram of Cit@AuNPs (d)

The TEM micrographs were used for examination of size, size distribution, and morphology of the Api@AuNPs and Cit@AuNPs (Figure 5). The average diameter of the Api@AuNPs is 19.1 ± 10.4 nm that represents the wider distribution in comparison with Cit@AuNPs (16.1 ± 5.7 nm). Despite repeated washing of Api@AuNPs, a marked point in these micrographs is the presence of an organic coating on the surface of the nanoparticles. Based on the outstanding stability of the Api@AuNPs in physiologic solutions and spectroscopic data, we concluded that this coating is API.

Similar TEM micrographs depict phytochemicals coating on metal nanoparticle surfaces that have been reported before [21, 22, 23, 24]. The Average hydrodynamic size of the nanoparticles was 27.5 ± 2.4 nm that higher than the acquired size from TEM studies. Similar hydrodynamic diameters were obtained for Cit@AuNPs (24.0 ± 4.2 nm). The zeta potential of the Api@AuNPs was −4.3 mV, and for Cit@AuNPs was −25.4 mV. We should note that unlike the Api@AuNPs, Cit@AuNPs were not resuspended in washing procedure that has been described in Section 2.2. Thus Cit@AuNPs represent higher negative charges. In our previous study, we report a gradual decrease in the zeta potential of CTAB coated gold nanoparticles through the similar procedure of washing that has been applied for Api@AuNPs [25].

### 3.2 Cell viability studies

**FIGURE 6:**
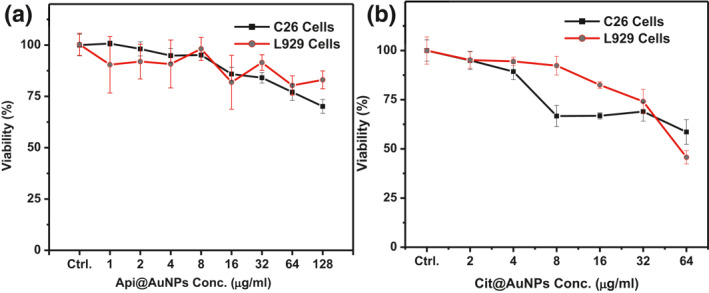
CT26 and L929 cells viability after 24‐h incubation with Api@AuNPs (a) and Cit@AuNPs (b) obtained by MTT assay

To apply Api@AuNPs in clinical cancer photothermal therapy, cytotoxicity of the nanoparticles was studied. MTT assay is regarded as a standard technique for the in vitro investigation of gold nanoparticles [26]. MTT assay represents very low toxicity on both CT26 and L929 cells with 24 h incubation of Api@AuNPs. Significant differences have appeared only at 128 μg/ml concentration (Figure 6a). Rajendran et al. report also show no cytotoxic effect of Api@AuNPs on normal epidermoid cells. However, almost 40% of epidermoid squamous carcinoma cells (A431) were killed by 24‐h treatment of 200 μg/ml of Api@AuNPs. Based on the TEM micrographs of A431, cellular uptake of nanoparticles has been shown in their report [9]. Based on Rajendran et al. and our results, the API molecule appears to be a good alternative to the citrate molecule in the synthesis of biocompatible gold nanoparticles.

Phytochemical‐coated metal nanoparticles are very biocompatible in comparison with chemical‐coated metal nanoparticles [7, 27]. Here we investigate the Cit@AuNPs cytotoxicity on the same cell lines up to 64 μg/ml. Our results show that even in a low concentration of Au, Cit@AuNPs is more toxic in comparison with Api@AuNPs (Figure 6). Since Cit@AuNPs are very unstable and cannot be concentrated by centrifugation, the pristine concentration of Cit@AuNPs was applied and higher concentrations of it were not investigated.

**FIGURE 7:**
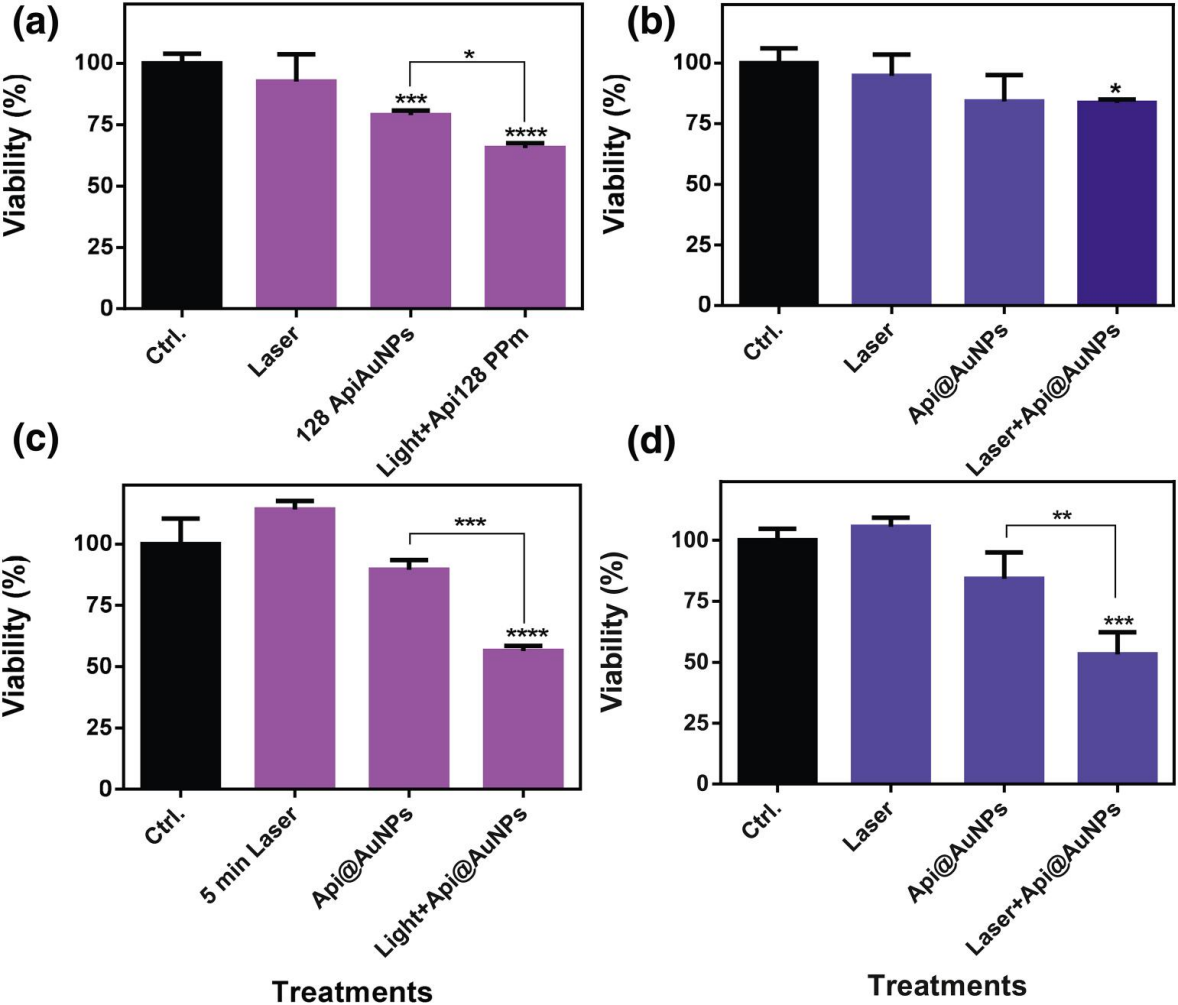
L929 (a), (c) and CT26 (b), (d) cell viability after 24 h of incubation with 128 μg/ml of Api@NPs and laser radiation the next day. (a) and (b) 3 min laser irradiation, (c) and (d) 5 min laser radiation. Values are displayed as the mean ± SD and were analysed using one way‐ANOVA (*p < 0.05)

Based on cytotoxicity results, 128 μg/ml concentration of Api@AuNPs was applied for PTT treatments. This concentration of Api@AuNPs plus 3 min light irradiation would kill almost 20% of CT26 cells and 30% of L929 cells, while 3 min light irradiation alone does not cause significant cell death in either of cell lines (Figure 7a,b). PTT treatment with 3 min of laser irradiation and 128 μg/ml concentration cause significant cell death only for L929 cell lines. Increasing the duration of laser irradiation up to 5 min leads to a significant increase in cell death, almost up to 50% in both cell lines (Figure 7c,d). This increase in cell death due to the increased laser irradiation time reflects the photothermal activity of the Api@AuNPs.

### 3.3 Apoptosis analysis

By utilizing plasmonic nanoparticles such as gold for the photothermal killing of cancerous cells, the usual cell death pathway is necrosis [2, 28]. Necrosis is a process that releases intracellular components into the extracellular environment inducing harmful inflammatory cytokines [29]. However, there are many factors that could impact the cell death pathway in the PTT process. Some of these factors are related to the irradiation condition such as laser energy [2] or type (pule or continuous‐wave laser) [30]. Some other factors depend on the type or properties of applied plasmonic particles [31].

**FIGURE 8:**
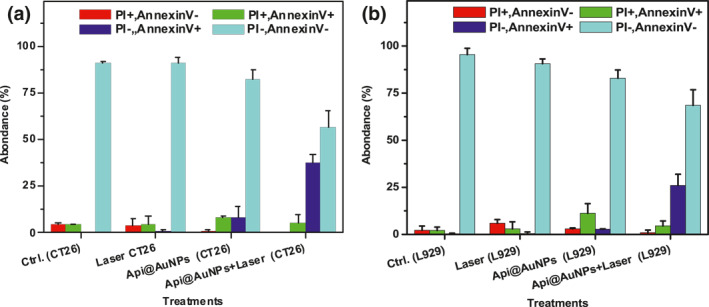
Assessment of cell death mechanism by Annexin V‐PI staining in CT26 (a), (b) and L929 (c), (d) cells after the separate and combined treatment of Api@AuNPs and laser (0.5 W/cm^2^, 5 min)

Based on Pattani et al. apoptotic rate of nanoparticles in PTT treatment depends on particle localization. A higher incubation time of the particles leads to more internalisation of the particles in the perinuclear environment of the cell and subsequent laser treatment leads to a higher value of apoptosis in the cell death pathway [32]. Based on previous studies on the subject, we have chosen an ideal condition for increasing the apoptotic rate of cell death in the PTT process. To evaluate the extent of apoptotic and necrotic percentage of cells, the flow cytometry examination was conducted. Figure 8 is a representation of the sum of the cytograms that were received for CT26 and L929 cell lines after each treatment. Diagram representing the bivariate PI/Annexin V examination of various groups. The viable cells have a negative value for both Annexin V and PI, while necrotic cells are PI‐positive, and apoptotic cells are Annexin V‐positive values [33].

Our study revealed that in both cell lines the apoptotic cell population is significantly higher on the groups that have been treated by Api@AuNPs mediated photothermal therapy in comparison with a single treatment of laser or Api@AuNPs (Figure 8). Rajendran et al. have declared that Api@AuNPs in higher concentrations (200 μg/ml) could solely trigger an apoptotic pathway [9]. However, here we observed a low population of apoptotic cells (8 ± 6%) in CT26 cells that have been treated by 128 μg/ml Api@AuNPs without light treatment. In our study, the apoptosis percentage of cells is 37.9 ± 4.3 in CT26 cells, and 26 ± 5.9 in L929 cells that is similar to the apoptosis rate that has been acquired for folate [34] or RGD [35] coated gold nanoparticles. It is necessary to keep in mind that bioconjugation of targeting moieties such as folate was performed for increasing the cellular uptake of the nanoparticles in which subsequently increased the apoptotic rate of the PTT process [36].

## 4 CONCLUSION

In this study, a natural flavonoid, apigenin, has been used for the synthesis of gold nanoparticles with an average diameter of 19.1 ± 10.4 nm (hydrodynamic diameter, 27.5 ± 2.4 nm). The nanoparticles have been washed through a series of centrifugation and decantation processes. The surface charge of Api@AuNP nanoparticles was confirmed to be −4.3 mV, which is much lesser than that of Cit@AuNPs (−25.4 mV). API coating was confirmed through FTIR and Raman spectroscopy investigation. Unlike Cit@AuNPs, Api@AuNPs are stable in the physiological environment and represent a much lower toxic effect on both CT26 and L929 cell lines. Based on the results, in vitro PTT treatment of cells with Api@AuNPs at a concentration of 128 μg/ml leads to the death of almost half of both cancerous and normal cells. Flow cytometry analysis revealed that the primary cell death mechanism is apoptosis. Based on the obtained results, Api@AuNPs could be replaced with Cit@AuNPs for various biomedical applications such as photothermal therapy treatment.

## CONFLICT OF INTERESTS

None.
